# Correction: 12 Novel clonal groups of Leptospira infecting humans in multiple contrasting epidemiological contexts in Sri Lanka

**DOI:** 10.1371/journal.pntd.0009471

**Published:** 2021-05-26

**Authors:** Dinesha Jayasundara, Indika Senavirathna, Janith Warnasekara, Chandika Gamage, Sisira Siribaddana, Senanayake Abeysinghe Mudiyanselage Kularatne, Michael Matthias, Jean-François Mariet, Mathieu Picardeau, Suneth Agampodi, Joseph M. Vinetz

The last author’s name was entered into the submission system incorrectly and therefore was indexed incorrectly on PubMed. The correct name is Joseph M Vinetz. The correct indexing in PubMed should read Vinetz JM.
