# Supplementary figures and images for: 12 Novel clonal groups of Leptospira infecting humans in multiple contrasting epidemiological contexts in Sri Lanka
Source: PLoS Negl Trop Dis. 2021 Mar 18;15(3):e0009272. doi: 10.1371/journal.pntd.0009272 (PMC8009393; doi:10.1371/journal.pntd.0009272)

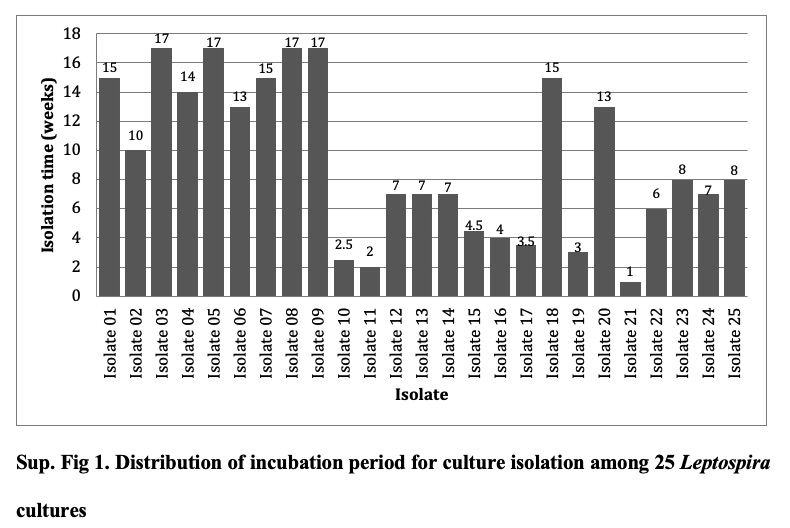

Supplement: S1 Fig — (JPG) [file pntd.0009272.s001.jpg]
